# Innovative solid desiccant dehumidification using distributed microwaves

**DOI:** 10.1038/s41598-023-34542-9

**Published:** 2023-05-06

**Authors:** Doskhan Ybyraiymkul, Qian Chen, Muhammad Burhan, Faheem Hassan Akhtar, Raid AlRowais, Muhammad Wakil Shahzad, M. Kum Ja, Kim Choon Ng

**Affiliations:** 1 BESE Division, Water Desalination and Reuse Center, King Abdullah University of Science and Technology, Thuwal, 23955 Saudi Arabia; 2 Institute for Ocean Engineering, Shenzhen International Graduate School, Tsinghua University, Tsinghua Campus, University Town, Shenzhen, 518055 China; 3 Department of Chemistry and Chemical Engineering, Lahore University of Management Sciences, Lahore, 54792 Pakistan; 4 Department of Civil Engineering, College of Engineering, Jouf University, Sakakah, 72388 Saudi Arabia; 5 Mechanical and Construction Engineering Department, Northumbria University, Newcastle Upon Tyne, NE1 8ST UK

**Keywords:** Energy science and technology, Engineering, Information theory and computation

## Abstract

Dehumidification is one of the key challenges facing the air conditioning (AC) industry in the treatment of moist air. Over many decades, the dual role of heat exchangers of AC chillers for the sensible and latent cooling of space has hindered the thermal-lift reduction in the refrigeration cycle due to the requirements of water vapor removal at dew-point and heat rejection to the ambient air. These practical constraints of AC chillers have resulted in the leveling of energy efficiency of mechanical vapor compressors (MVC) for many decades. One promising approach to energy efficiency improvement is the decoupling of dehumidification from sensible processes so that innovative but separate processes can be applied. In this paper, an advanced microwave dehumidification method is investigated in the laboratory, where the microwave (2.45 GHz) energy can be irradiated onto the dipole structure of water vapor molecules, desorbing rapidly from the pores of adsorbent. Results show a significant improvement in performance for microwave dehumidification, up to fourfold, as compared to data available in the literature.

## Introduction

**Figure 1:**
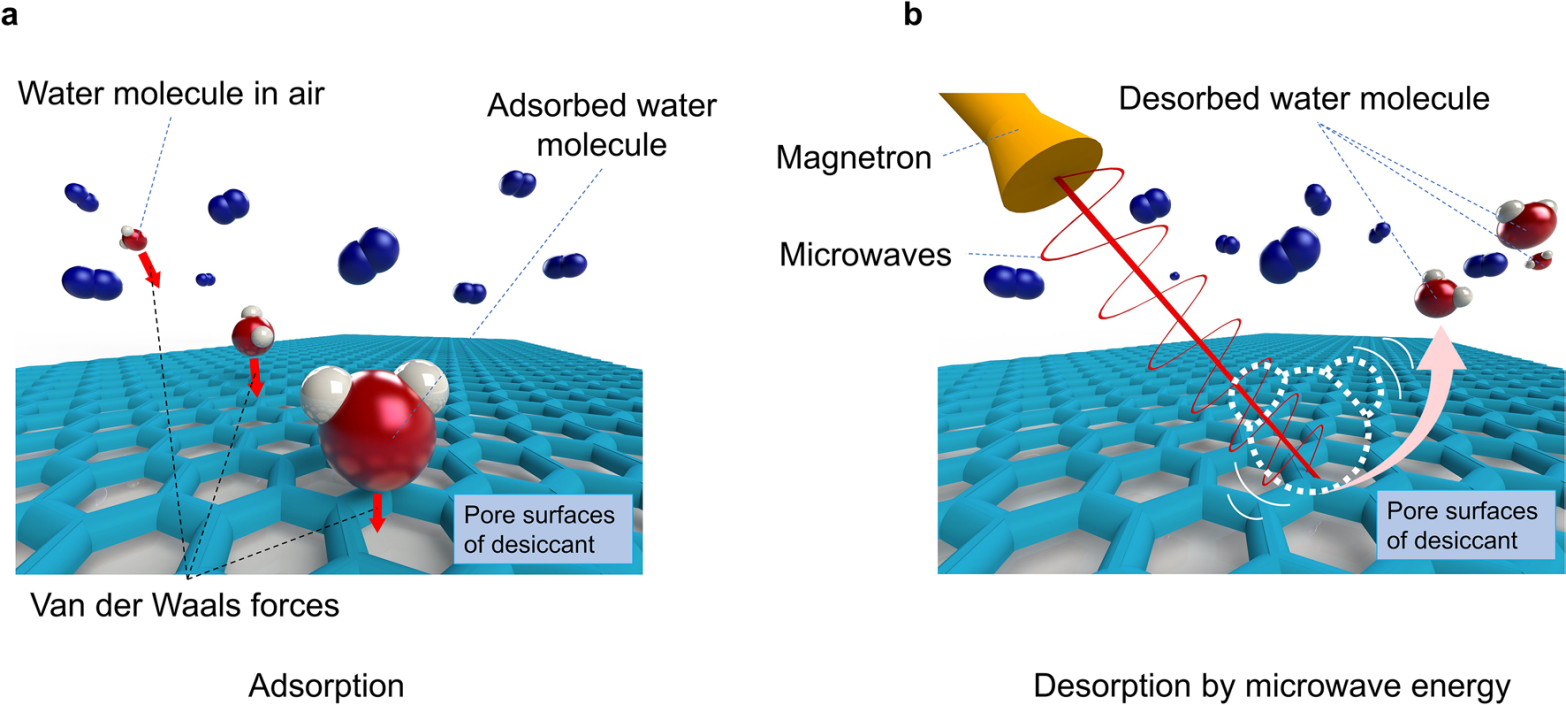
Schematically representation of microwave dehumidification. (**a**) Dehumidification of airstream by adsorption of water vapor from the moist air onto the pore surface of the adsorbent due to Van der Walls forces. Nitrogen and oxygen molecules in the air are very little attracted at ambient pressure and temperatures; (**b**) Desorption of water vapor from pores of adsorbent due to irradiation by microwave energy, where microwave energy (energized by oscillating) is directly delivered to polar adsorbed water molecules. Desorbed water molecules and air molecules are almost not adsorbing microwave energy as they can freely move in a gas state. Microwave desorption is needed to regain the adsorbent's ability to attract water molecules.

Dehumidification is water vapor removal from the air to maintain human comfort and a healthy environment (relative humidity (RH) at 40%-60%)^1–4^. Presently, dehumidification is provided by cooling the airstream to its dew-point to condense water vapor using a dual-role AC chiller^5^; and air-cooled AC chillers have reached their asymptotic performance limit, 0.7–0.85 kW/Rton (equivalent to a coefficient of performance (COP)^6^ of 4–5)^7^. Much literature on chiller manufacturers cites low kW/Rton is attributed to acceptance test conditions ignoring electricity consumption incurred by long chilled water piping losses^5^. One of the solutions to improve the performance of AC is to decouple dehumidification from sensible cooling, thus permitting the incorporation of new dehumidification methods. It is well known that microwaves can desorb water molecules from adsorbent or sorbent. Therefore, the mechanism is used in microwave dehumidification, which is an emerging environmental-friendly method. In microwave dehumidification, the air becomes dehumidified due to the attraction of water molecules onto a solid adsorbent (desiccant) pore surface by physio-sorption (physical adsorption)^8–11^, a characteristic of weak Van der Walls forces^12–15^. When adsorbent pores are saturated with water, microwave-assisted desorption (removal of water) initiates, and highly humid air purges out. The process is schematically presented in Fig. 1a,b.

From the available literature, Roussy and Chenot demonstrated the first microwave dehumidification process with a single-mode waveguide in 1981^16^. They presented the dependence of desiccant temperature on the electric field^16^. Moreover, Roussy et al. proposed a model to represent the fast kinetics of microwave desorption^17^. Most of the research within 40 years has been focused on developing the microwave-assisted desorption method with small volumes^16–22^. Notably, the investigation was extended with different adsorbents (activated alumina, zeolite, and silica gel)^18^. Many advantages of microwave desorption were revealed, such as transferring energy more efficiently than convection energy transport^23^ and desorbing at low temperatures due to direct energy transport^24^. However, a critical parameter such as COP was usually omitted in the literature. In addition, no electrical power values were provided; instead, microwave power was shown. Therefore, the microwave coefficient of performance (MCOP) was introduced, which can be the platform for comparing different microwave dehumidification systems. MCOP can be calculated using microwave power, microwave exposure duration, and water desorbed amount. The calculated values of MCOP for other authors were extremely low, as summarized in Table 1. The system's performance depends on the uniform propagation of the electric field^25^, the geometry of the microwave chamber, microwave irradiation time, the type of irradiation (continuous, pulsed), and the reflected power amount. A multi-mode chamber system similar to a home oven was used for desorption^19^. Furthermore, the fixed zeolite-coated desiccant rotor was regenerated using microwave and temperature swing desorption methods, but the performance was low, with MCOP around 0.22^21,22^.

**Table 1:** A comparison of microwave dehumidification systems that are available in the literature. MCOP refers to the ratio of desorbed enthalpy of water vapor to irradiated microwave energy. MCOP values were calculated or obtained from the literature. Calculation of MCOP for literature is provided in supplementary materials.

References	MCOP	Irradiation duration (min)	Desorbed water mass (g)	Microwave energy (kJ)
Roussy et al.^17^	0.09*	1.66	0.67	17.5
Polaet et al.^18^	0.018*	130	2.73	390
Kubota et al.^22^	0.22	–	4	47

*calculated values.

Despite many decades of studying microwave dehumidification, we have discovered a knowledge gap that hindered its development. It is associated with narrowing research focus on methods advancement with small samples over the past half-century. The bounding of research by small single-mode cavities is due to the uniform electric field distribution, which led to impeding factor neglect, such as low performance. So far, most experiments have been carried out on scales less than 160 g with a volume of 1 L^16–22^. Consequently, all bench-scale studies were not developed to the pilot level to dehumidify the air. Scaling up the bench-scale system can lead to an uneven distribution of the electric field, heating of narrow sections (close to the surface), and, consequently, a drop in efficiency^25^. For achieving a high-performance pilot system, the following critical limitations had to be solved: (I) Internal entropy generation due to unheated areas (the non-uniformity of the electric field); (II) Enormous energy wasting (a high reflection of microwaves power); (III) Excess microwave irradiating time.

In this paper, we experimentally demonstrated that 97–99.5% of irradiated microwave power could be efficiently utilized, increasing performance by fourfold at a laboratory-scale pilot for the first time. Optimization allowed us to reduce a reflected power to 0.5–3% of input microwave power and distribute microwaves homogeneously. The key novelties are as follows: (a) A rotating reflector with attached honeycomb desiccant was proposed; it makes a more uniform distribution of the electric field and prevents overheating of desiccant; (b) a new approach of optimization was proposed to decrease reflected microwave power and unheated areas; (c) The effects of microwave irradiation time and heat recovery on COP were experimentally evaluated. The proposed dehumidification system with an optimized structure and operating parameters will help overcome all of the above limitations and achieve sustainable green dehumidification goals. We hope our work will help further model microwave processes and develop emerging technologies. A detailed explanation of the system is provided in the following sections.

## Results

**Figure 2:**
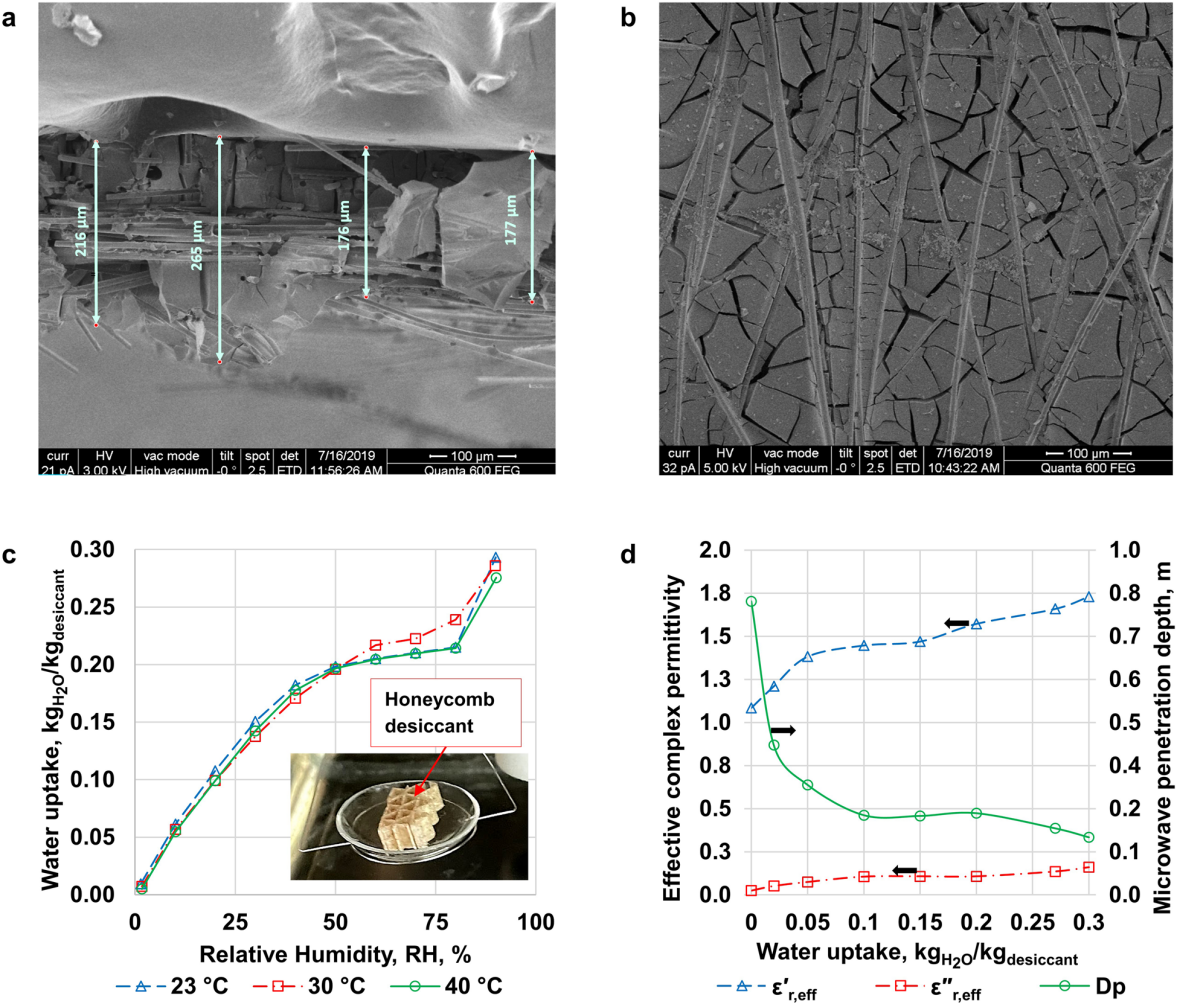
Characteristics of composite (honeycomb cellulose, adsorbent, and binder) desiccant structure. (**a**) SEM image of desiccant cross-section; (**b**) SEM image of desiccant surface; (**c**) Isotherms of composite desiccant structure. A composite desiccant sample on the crucible of dynamic vapor sorption analyzers "Aquadyne DVS"; (**d**) Dependency of effective complex permittivity and penetration depth of microwaves on adsorption water uptake at 2.45 GHz.

Microwave dehumidification was developing with negligence of performance hitherto, so numerical optimization and experiments were highly focused on performance. The RD-type silica gel-coated desiccant wheel with a honeycomb structure was used as a desiccant wheel. The diameter of the wheel was 0.448 m, the height was 0.4 m, the composite material density was 668 kg/m^3^, the volume, including voids, was 0.063 m^3^, and the dry mass of the desiccant wheel was 11.8 kg. The thickness of honeycomb channel wall's average thickness (including coating + cellulose + binder) was obtained from a cross-sectional SEM image (thickness was 208 µm), which is shown in Fig. 2a. Figure 2b depicts a fractured desiccant coating surface bound with fiber material. These fractures increase mass diffusion and flow of moisture. The selection of honeycomb structure and RD-type silica gel was due to its high-water adsorption capacity and high microwave penetration depth. Additionally, adsorption isotherms of the desiccant wheel, which is a combined (honeycomb cellulose, adsorbent, and binder) desiccant structure, were obtained, as shown in Fig. 2c. According to the results, the composite structure can adsorb moisture up to 30% of its dry bone mass at higher humidity. However, it can adsorb around 20% of its dry bone mass within a moderate humidity region. Besides, Fig. 2c demonstrates a composite desiccant sample on the crucible of the dynamic vapor sorption analyzer "Aquadyne DVS," which operates on the gravimetric principle and is fully automated for measuring adsorption isotherms. An Agilent impedance analyzer was used to measure the complex permittivity of composite desiccant material that was uniformly sampled without voids, and then effective values were determined according to Eq. (2). Figure 2d illustrates the complex permittivity (dielectric properties) of a composite desiccant material with different adsorption uptakes. Penetration depth is the depth at which the electric field is reduced to 37% of its entrance value in the medium. It should be noted that microwaves can penetrate to the center of the desiccant wheel as the wheel's radius (0.224 m) is lower than the penetration depth at the operating range of adsorption uptake (0.05–0.2). As the amount of adsorbed water reduces, the microwave's penetration depth is increased with decreasing in the desiccant wheel's complex permittivity. Moreover, it reveals that the penetration depth varies little at the operating range of adsorption uptake (0.05–0.02), which helps in simulation simplification.

### Chamber design and optimization

**Figure 3:**
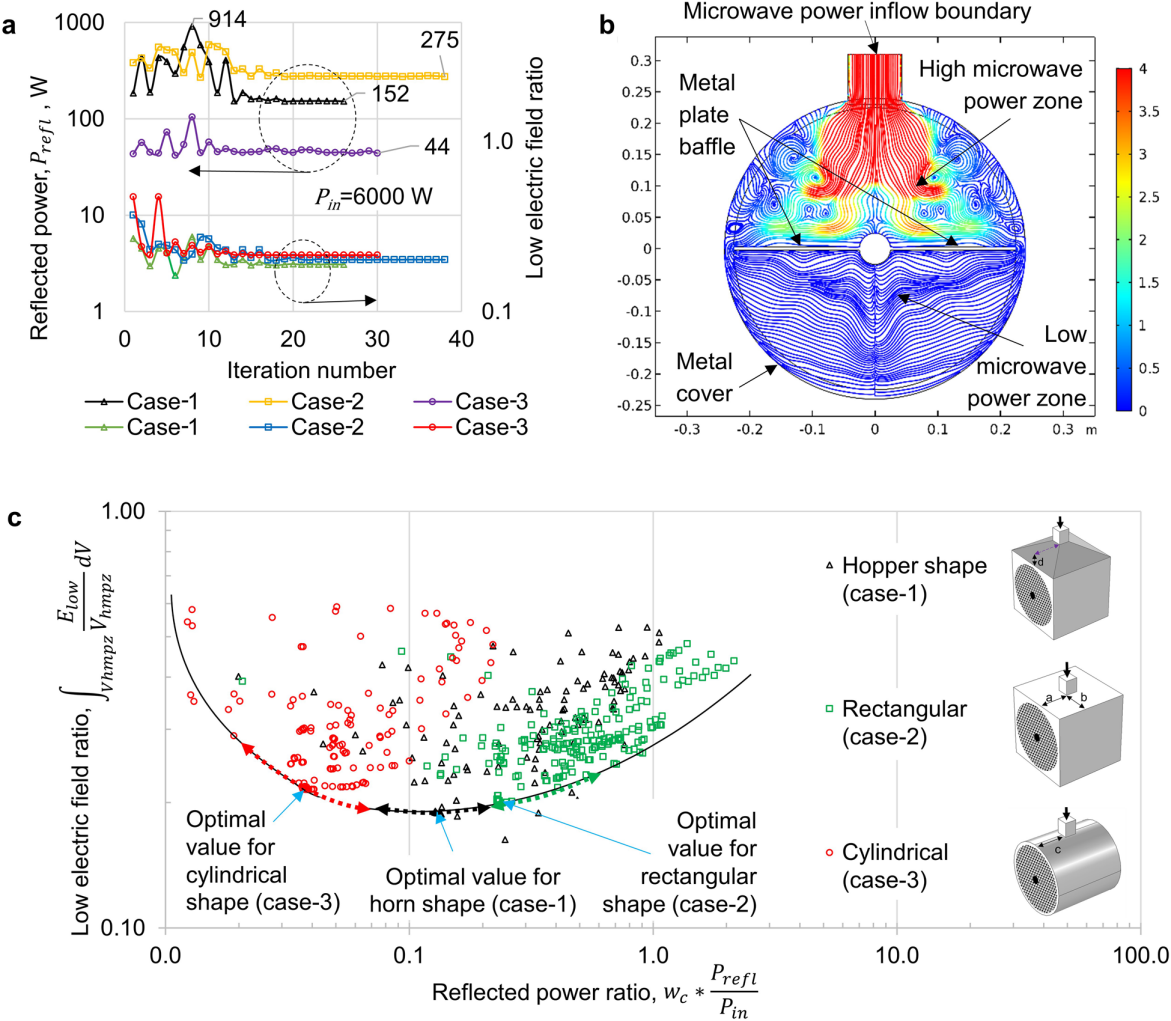
Optimization results of a microwave dehumidification system. (**a**) Reflected power and low electric field ratio for the most optimal 3 cases of global optimization with random initial control variable; (**b**) Streamline of the “Poynting” vector of microwaves at the cross-section. The desiccant wheel is embedded in the cylindrical microwave chamber. The wavelength of the microwave is 0.122 m. The radius of the cylinder is r = 0.5 m. The color scale refers to the normalized value of S. (**c**) Objective functions space for multi-objective global optimization with random initial control values.

Three global optimizations were done for 3 cases (cylindrical, rectangular, and hopper shape) with random initial control variables, as explained in the section “Methods” (subsection “Multi-objective optimization of the microwave chamber”). Figure 3a shows the evolution of reflected power and low electric field ratio for the most optimal 3 cases of global optimization with a random initial control variable. Moreover, within 3 cases, case-3 was the most optimal. The optimum value of the control variable (illustrated in Fig. 6b) for case-3 was equal to 0.14 or c = 0.14. The difference in low electric field ratio was insignificant in 3 cases at optimal values. Results show that microwave emitting waveguide should be placed at the central part of the chamber than aside to get a more homogeneous (uniform) propagation of electric field, which corresponds to the lowest electric field ratio. The reflected power for case-3 is 44 W (0.7% of emitted power), which is the lowest value among other cases. The low reflection cannot be explained by the position of the waveguide as the reflected power increases during optimization to 914 W (15% of emitted power). Nevertheless, circular geometry had the lowest microwave power reflection during the optimization. Optimization results demonstrate that the shape of the chamber has a significant impact on performance than the position of the waveguide. Figure 3b illustrates streamlines of the Poynting vector of microwaves at the cross-section of the chamber. These streamlines indicate the propagation path of microwaves. The color scale refers to the normalized value of S (power flow). According to the results, most of the microwave power was adsorbed during the reflection (bouncing) of the microwaves inside the chamber. Owing to the parabolic form (which can be seen at the cross-section), the metal cover of the chamber in case-3 prevents returning of microwave rays back to the magnetron. Figure 3c shows objective function space, where we can also spot the optimum values for 3 cases located near the optimum front line, which is also called the Pareto front. The sum of objective functions at case-3 (cylindrical shape) reached extremum value, so the global optimum condition belongs to case-3 with c = 0.14 m.

### Experimental results

Based on the optimization results, the system with an efficient multi-mode chamber was built by the authors. Experiments were carried out in two modes: without heat recovery and with heat recovery (from the outflow of purged air).

**Figure 4:**
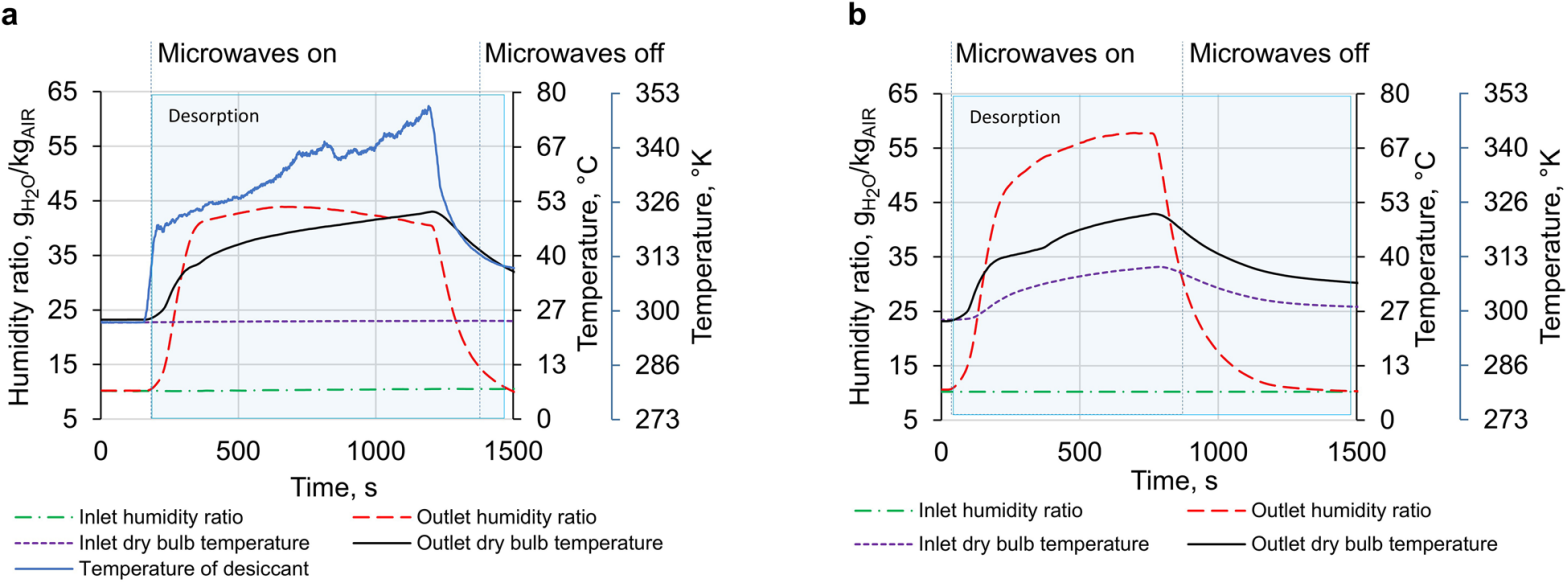
Experimental results of a microwave dehumidification system. (**a**) Humidity ratio and temperature for non-heat recovery mode; (**b**) Humidity ratio and temperature for heat recovery mode. Experimental results correspond to the maximum COP value of each mode.

The most efficient one (maximum COP) within the experimental results (different microwave irradiation, air flow rate) were demonstrated in Fig. 4 a,b for each mode. Figure 4a shows temperature and humidity ratio profiles at the inlet and outlet of the system without heat recovery. Microwave radiation time was equal to 17 min. However, the desorption time was longer than the radiation time due to the residual energy (thermal mass of the desiccant wheel). Inlet air temperature was constant during the adsorption and desorption cycles, and it was equivalent to 24 °C. Similarly, the inlet humidity ratio (ω) was equal to 10.3 g/kg throughout the experiment. As presented in Fig. 4a, the temperature of the desiccant wheel increased sharply at the start of microwave emission. The outlet air temperature increased over time, but its temperature was lower than the wheel temperature.

It proved that energy was transported by microwaves directly to adsorbed water. Consequently, pressure on the surface of the adsorbent increased, raising the outlet value of the humidity ratio to 43 g/kg. Nevertheless, the humidity ratio started declining after 500 s and reached 40 g/kg at the stopping time of microwave irradiation. As the airflow rate during the desorption was constant and equal to 185 m^3^/h, decreasing the outlet humidity ratio evidently reduced the system's performance. The outlet humidity ratio was meager due to the thermal mass of adsorbed water and desiccant at the starting time of microwave emission. The thermal mass effect needs more microwave emission time. However, the decreasing trend of the humidity ratio demonstrates that it cannot be very long. 2 kg of water was desorbed for the current mode during the desorption cycle, showing that many water vapors can be captured and turned into potable water or used to run IEC systems. The COP of the system was 0.55, and MCOP was 0.83. The temperature of the desiccant wheel did not go very high, which proves the excellent distribution of the electric field obtained by numerical results. Hot spots or decreasing performance of the system were not observed due to the continuous operation of the stirrer at the center of the desiccant wheel and wheel rotation that made the system safe and sustainable. Moreover, the desiccant temperature did not exceed 80 °C. Nevertheless, some transported energy is wasted by heating the outlet temperature to 51 °C, so it is motivated to consider the mode with heat recovering from hot outlet air to inlet air by the heat exchanger. Figure 4b presents temperature and humidity ratio profiles for microwave desorption with a heat recovering mode.

**Figure 5:**
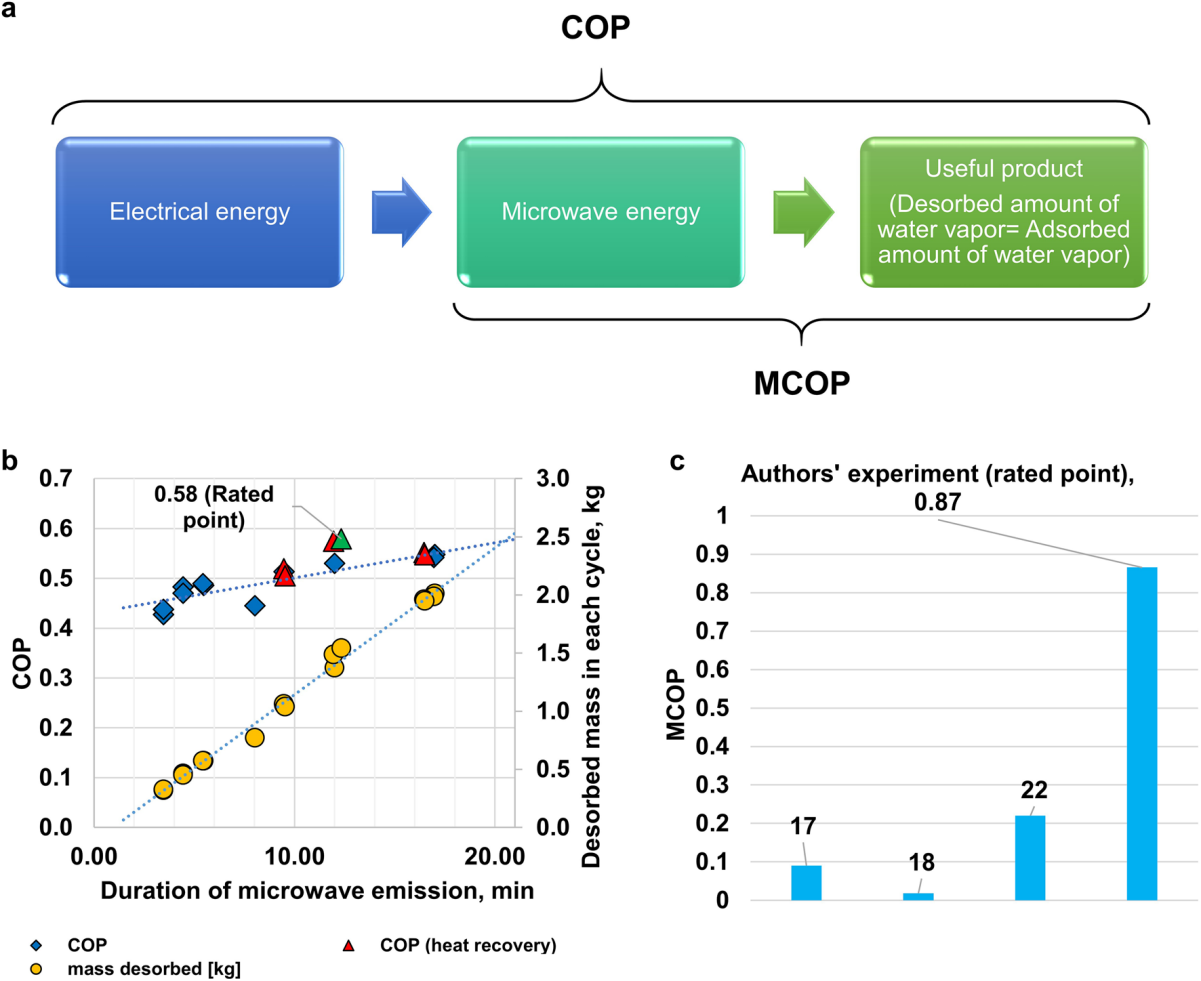
Performance of microwave dehumidification system. (**a**) Methodology for calculation of MCOP to compare the efficiency of different authors' microwave dehumidification systems and their difference from COP calculation concept. (**b**) COP and desorbed amount of water vs. duration of microwave emission. (**c**) Comparison of dehumidification performance with reference authors with current advance, which is 4 times higher than other authors.

A schematic diagram of the mode is shown in Fig. 7b. Microwaving time was equal to 12 min 20 s, and the airflow rate was 140 m^3^/h. Inlet air temperature increased due to heat recovering from the outlet temperature. Besides, the temperature profile was different from the temperature profile without heat recovery; notably, the outlet air temperature reached 51 °C in a shorter time than the previous mode. As a result, the system has the highest COP than other modes, and COP is equal to 0.58, and MCOP is equivalent to 0.87. Moreover, high performance can be seen from the humidity ratio profile that increased with time until microwave irradiation was stopped. Compared with the non-heat recovering mode, some wasted heat is used efficiently, thus increasing system performance. Around 1.54 kg of water vapor was desorbed from the desiccant wheel during the desorption process. Figure 5b demonstrates system performance and amount of desorbed water for the different duration (3.5–17 min) of microwave emission for both modes. The desorbed amount of water had almost linear dependence from time. Results show that COP increases with the duration increasing of microwave irradiation for non-heat recovery modes because of the thermal mass of saturated composite desiccant. At the beginning of microwave radiation, some portion of energy was used for rapid heating of the saturated desiccant wheel from 24 °C to 48 °C (Fig. 4a), so COP at the short time was low. Running the microwave longer, we can reduce the effect of thermal mass and increase the COP of the system. However, microwave irradiation was not more than 17 min as most of the water was desorbed (adsorption uptake was 0.03).

## Discussion

Not focusing on the performance of microwave dehumidification was the gap in research hitherto. Since simulation and experiment were targeted at getting the maximum performance, the most efficient microwave dehumidification system was developed with COP of 0.58 and MCOP of 0.87. MCOP of 0.87 means most of the microwave energy was delivered to water molecules directly, with the least internal entropy generation and low reflected power to the magnetron. Internal entropy generation is attributed to uneven heating within the volume. It occurs in high values region of “low electric field ratio.” Reflected microwave power was at the lowest value (0.5–3% of input microwave power), which has a tremendous impact on microwave dehumidification performance. The microwave generator's conversion efficiency, which turns electrical energy into microwave energy, was 0.7, which was accountable for the COP of 0.58 when MCOP was as high as 0.87. However, COP was the highest within the available literature. The highest COP (0.58) for heat recovery modes corresponds to the time when the humidity ratio reaches the highest value. Recovering heat can increase performance, but heat recovery has less effect for a short time or a long time. Meanwhile, the desorbed amount of water for heat recovery modes was more elevated than for non-heat recovery modes. The performance of microwave desorption should be considered as the general coefficient of performance (COP) and microwave power-based coefficient of performance (MCOP) by the following equation:

1 $$MCOP = \frac{{\Delta m*h_{fg} }}{{E_{mw} }}$$

2 $$COP = \frac{{\Delta m*h_{fg} }}{{P_{elec} }}$$

where $\Delta m$ is desorbed (water) moisture mass in [kg], ${h}_{fg}$ is a heat of evaporation in [J/kg], ${E}_{mw}$ is microwave energy emitted from the magnetron in [J], and ${P}_{elec}$ is electrical energy consumed in [J]. Dehumidification by desiccant always works as a cycle (adsorption and desorption), so the amount of adsorbed and desorbed water mass are the same. To focus on the performance of microwave dehumidification in the calculation, desorbed water mass was used. Hence the conversion efficiency $\eta$ is 0.7, Fig. 5a illustrates the difference between MCOP and COP in the methodology of estimation. Figure 5c shows a comparison in MCOP for different authors with microwave desorption. It can be seen that the current system that was developed, designed, and built by authors at King Abdullah University of Science and Technology (KAUST) has the highest COP and MCOP. It verifies that numerical optimization of chamber shape and obtaining the best operating conditions can overcome both limitations. Quantum jump was achieved in microwave dehumidification, with a fourfold increase in the MCOP, up to 0.87, as compared with available literature. Improving the microwave-based MCOP allowed us to cross-compare the microwave performances of assorted authors. Another issue was not considering microwaving time on the performance of the system.

The system was improved to commercial large system performance. It shows that building a microwave system needs an understanding of the physics and mechanics of electromagnetic waves, which can help design a system with high safety standards and high performance. The role of numerical optimization methods is crucial, as well as experiments.

## Conclusion

A laboratory-scale microwave dehumidifier pilot was successfully tested to achieve a fourfold improvement in the MCOP up to 0.87 compared with available literature. These dehumidification improvements were attributed to the better waveguide and chamber design, demonstrating the sensitivity of water molecules desorption (dehumidification) to microwave energy delivery. However, the parasitic losses of electricity conversion to microwave, around 30% of electricity input, have resulted in an overall COP of 58%. Hence, there is much room for the electrical technology of microwave generation research, should a sustainable microwave dehumidifier COP of 75% is desired.

## Methods

### Theory of microwave dehumidification

Van der Waals force (or energy sites) attract water molecules onto the surface of sold desiccant material during dehumidification (Fig. 1a). Adsorbed water can be removed by pressure swing^26,27^ and thermal swing^12,28^. It is the most energy-consuming process in dehumidification^29^. On the other hand, attraction forces have electrostatic behavior, so oscillating dipole-structured water molecules with electromagnetic waves (microwaves) could detach from the surface faster and with less energy compared to the above-mentioned methods. Microwave-assisted desorption is an emerging method (Fig. 1b), where two desorption mechanisms are applied: the direct microwave effect on molecules (selective energy transport) and the thermal microwave effect^23^. It does not require heating of purged air stream to transport energy as in thermal swing; instead, the energy is transported directly to the water molecule^23^. Microwaves are electromagnetic waves ranging from about 1 m to 0.001 m (with frequencies between 0.3 GHz and 300 GHz)^30^, and like all electromagnetic waves, it obeys Maxwell's equation systems. The time-harmonic electromagnetic field can be represented by the following differential equation that is obtained from Maxwell equation systems by applying a frequency-domain approach:

3 $$\nabla \times \left( {\nabla \times \vec{E}} \right) - k_{0}^{2} \left( {\varepsilon_{r,eff}^{ } } \right)\vec{E} = 0,$$

where $\nabla$ (nabla) is a vector differential operator, $\overrightarrow{E}({E}_{x},{E}_{y},{E}_{z})$ is the vector field of an electric field in [V/m], ${k}_{0}$ is wavenumber in [rad/m]. ${\varepsilon }_{r,eff}$ is effective complex permittivity, and it has real and complex components, as shown by the following equation:

4 $$\varepsilon_{r,eff} = \varepsilon_{r,eff}^{^{\prime}} + i\varepsilon_{r,eff}^{^{\prime\prime}}$$

where ${\varepsilon }_{r,eff}^{"}$ is the real part of effective complex permittivity (dielectric constant), ${\varepsilon }_{r,eff}^{"}$ is the imaginary part of effective complex permittivity (dielectric loss factor). In simulations, averaged microwave power consumed by the desiccant wheel is calculated according to the Poynting equation: ${P}_{mw}=V\pi f{\varepsilon }_{0}^{ }{\varepsilon }_{r,eff}^{"}{E}^{2}$, where* P*_*mw*_ is the microwave power in [W], *V* is the desiccant wheel's volume in [m^3^], *f* is the microwave's frequency in [Hz], ${\varepsilon }_{0}$ is the free space permittivity in [F/m]. Another important parameter is the time-averaged vector field ($\overrightarrow{S}$), which showed the power flow and microwave direction. $\overrightarrow{S}=\overrightarrow{E}\times \overrightarrow{{H}^{*}}$, where $\overrightarrow{{H}^{*}}$ is the vector field of the magnetic field and complex conjugate. Silica gel was chosen among adsorbents and coated on a cellulose-based honeycomb structured wheel to achieve a high surface area per unit volume of the wheel. These materials (silica gel and cellulose) are almost transparent to microwave radiation; hence, microwave energy is solely focused on ejecting the water molecules from the pores of the adsorbent.

### Effective complex permittivity and penetration depth

The honeycomb-based adsorbent wheel permits airflow through its channeled voids. For accurate modeling, it is necessary to obtain the effective complex permittivity of the honeycomb wheel, which is a function of complex permittivity of air and desiccant materials (silica gel, binder, cellulose), that is^31^:

5 $$\varepsilon_{r,eff}^{ } = f_{op} \varepsilon_{r,air}^{ } + f_{cd} { }\varepsilon_{r,cd} = f_{op} \left( {\varepsilon_{r,air}^{^{\prime}} } \right) + f_{cd} \left( {\varepsilon_{r,cd}^{^{\prime}} + i\varepsilon_{r,cd}^{^{\prime\prime}} } \right)$$

where *f*_*op*_ is the volume fraction of air in the openings (honeycomb), and *f*_*cd*_ = *1-f*_*op*_ is the volume fraction of composite desiccant. The penetration depth of microwaves is also calculated with effective complex permittivity by the following formula^23^:

6 $$D_{p} = \frac{{\lambda_{0} }}{{2\pi \sqrt {\left( {2\varepsilon_{r,eff}^{^{\prime}} } \right)} }}\frac{1}{{\sqrt {\left( {\sqrt {1 + \left( {\frac{{\varepsilon_{r,eff}^{^{\prime\prime}} }}{{\varepsilon_{r,eff}^{^{\prime}} }}} \right)^{2} } - 1} \right)} }}$$

where ${D}_{p}$ is the penetration depth of microwaves in [m] and ${\lambda }_{0}$ is the wavelength of microwaves in [m].

### Multi-objective optimization of the microwave chamber

**Figure 6:**
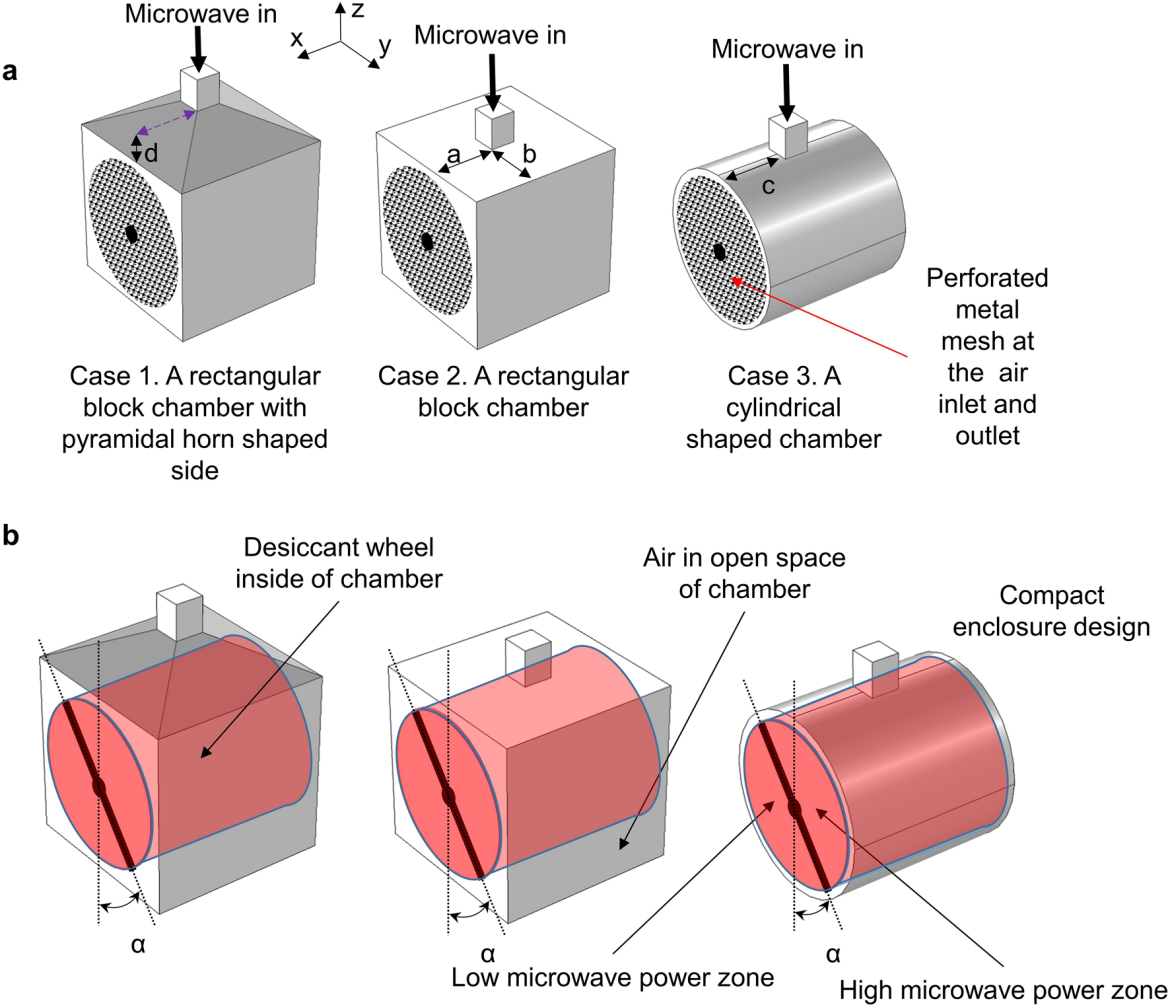
Numerical optimization domains and geometry of microwave chamber. (**a**) Microwave chambers with 3 different shapes, namely, a rectangular block shape with a pyramidal hopper-shaped side, a rectangular block shape, and a cylindrical shape. Microwave emitting waveguide positions variables a,b,c,d. which are used as control variables in optimization; (**b**) Positioning of the desiccant wheel in the 3 different chambers, where the air is colored gray, and the desiccant wheel is colored red to show different domains with different properties for simulation in 3 cases.

Optimization was conducted, minimizing the reflected microwave power and unheated areas to increase heating performance within the honeycomb wheel. Three multi-objective optimizations with the weighted sum of objectives were carried out as three shapes were considered, namely, (i) the first is a rectangular block chamber with a pyramidal hopper-shaped side, (ii) the second is a rectangular block, and (iii) a cylindrical chamber. The control variables were denoted by a,b,c,d and displayed in Fig. 6a. Global optimum value was obtained with random initial control values within the constraining range. Moreover, the optimization was constrained by the chamber's geometry, with wheel dimensions kept constant at a radius of 0.224 m and height of 0.4 m (Fig. 6b). For mathematical modeling, the following assumptions were used: 1) The complex permittivity and the effective complex permittivity of honeycomb material are homogeneous and isotropic; 2) The perforated metal sheet was assumed to have the same reflective characteristics as the non-perforated one due to the much smaller perforation hole diameter (4 mm) than the wavelength of microwaves (124 mm). For the design of the waveguide and chamber, Eq. (1) was solved to obtain the electric field (V/m) subjected to the boundary conditions. At the entrance of the waveguide (from the magnetron), the electric field of the x-direction is designed according to Eq. (7) whilst the corresponding values in the y and z directions are zero^32^:

7 $$E_{x} = \sqrt {P_{in} /P_{mode} } { *}sin\left( {\left( {w - y} \right)/w{*}pi} \right)/w,\;E_{y} = 0,\;E_{z} = 0.$$

Equation (7) would satisfy the microwave irradiation classified as the TE_10_ mode under the standard industrial waveguide (WR340) at a frequency (*f*) of 2.45 GHz. The assumption of the perfect electrical conductor was applied for all walls and perforated sheets, where tangential components of the electric field were set equal to zero:

8 $$\vec{n} \times \vec{E} = 0$$

The computation region consists of two domains (Fig. 6b) because air (gray) and the desiccant wheel (red) have different effective complex permittivity. The Nelder-Mead algorithm was used for optimization calculations. Nelder–Mead algorithm is a nonlinear optimization method that uses the simplex concept. At each iteration, a new vertex is defined by the four operations known as reflection, expansion, contraction, and shrinkage. The value of the objective function at n + 1 vertex of a simplex is calculated as it is moved toward the minimum point^33^. Two objective functions were defined such as reflected power ratio and low electric field ratio:

9 $$f_{p} = w_{c} *P_{refl} /P_{in} \;{\text{and}}\;f_{low} = \mathop \smallint \limits_{Vhmpz}^{ } E_{low} dV/V_{hmpz}$$

where the weighting coefficient (${w}_{c}$) was equal to 5 as usually 20% of microwave power was reflected back.

10 $$E_{low} = \left\{ {\begin{array}{*{20}l} {1,\quad if\left( {E_{norm} < E_{threshold} } \right);} \\ {0,\quad {\text{ otherwise}},} \\ \end{array} } \right.$$

where ${E}_{threshold}$ was equal to 3000 V/m, which was discovered from the authors' waveguide experiment. A low electric field ratio is needed to maintain uniform heating by microwaves. Control variables and their bounds for all cases are presented below:

Case-1: 0.5[m] > h > 0.005[m]; Case-2: 0.2[m] > a > 0, 0.25[m] > b > 0; Case-3: 0.2[m] > c > 0.

COMSOL Multiphysics computational platform was used to perform optimization. The system of equations was solved with FGMRES Iterative Solver, which uses the restarted flexible generalized minimum residual method. Mesh built of (minimum) 2,251,507 tetrahedral domain elements and 73,048 triangular boundary elements.

### Experimental apparatus

A microwave generator (Fricke und Mallah, Germany) with an efficiency value of 0.7 for converting alternative current (AC) electrical (9 kW) power to microwave power was used to generate the microwaves. A magnetron head (with a circulator and a directional coupler) was used to generate microwaves and measure forwarded and reflected microwave power. A three-stub tuner (Fricke und Mallah, Germany) was used to tune the microwave phase to perform impedance matching for maximizing energy transfer. Frame and equipment were grounded with protective grounding to prevent users from high voltage electrical hazards.

A specially made double Faraday cage was used in order to avoid microwave leakage to the surrounding area. The first cage is a multi-mode chamber where a desiccant wheel was placed, and its geometry was built based on numerically optimized results (more detailed information about numerical optimization is provided in Sects. 4 and 5). The first cage was placed in the second cage, and both cages are made of a 3 mm thick aluminum sheet. Aluminum has low resistance and high microwave reflective characteristics. An electrical motor (DKM motors, Korea) was used to rotate a desiccant wheel with a preset speed to control the microwave process. A three-phase electrical power meter PowerLogic PM5110 (Schneider Electric, UK), with a measuring range of 3–30 kW and with (0.5% FS) accuracy, was used to measure electrical power. A calibrated aluminum nozzle was used for airflow measurements that were made according to the ISO/ANSI standards with high accuracy (RecoV, Italy).

A differential pressure sensor Model 264 (Setra, USA) with high (± 0.25% FS) accuracy and with a range of 0–250 Pa was used to measure differential pressure across the nozzle. Pt100 RTD temperature sensors (Omega, UK) were used to measure dry and wet bulb temperatures with an accuracy of ± 0.1 °C and with a range of − 20–350 °C. An infrared temperature sensor OS-MINIUSB (Omega, UK) with an accuracy of 1 °C was used to measure a rotating desiccant wheel temperature that can measure at the range of 0–250 °C. An automated data logging system was created by authors on the software LabVIEW that logs data from Agilent 34970A. An air heat exchanger was used to recover energy from outlet air, and air-flowing channels, ducts, and microwaving chamber were thermally insulated with foam rubber. A microwave leak detector was running all the time for safety reasons.

**Figure 7:**
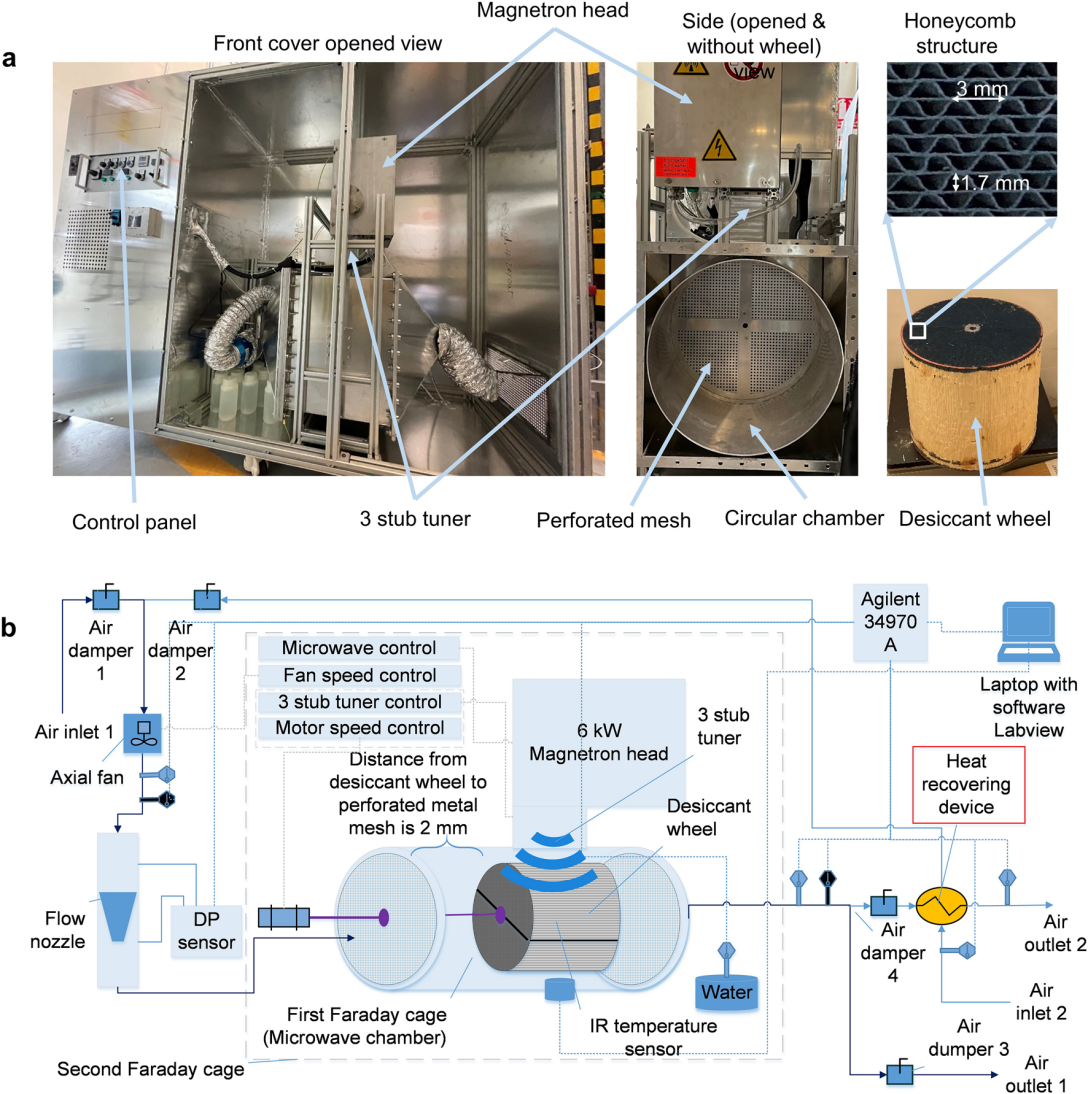
View of experimental microwave dehumidification pilot system. (**a**) Pictorial view of the microwave dehumidification system, composed of the control panel, magnetron head (where microwaves are generated), 3-stub tuner, microwave chamber, and desiccant wheel. Enlarged images show pitch in honeycomb structure; (**b**) Schematic diagram of the microwave dehumidification system. Heat recovery is affected by a heat exchanger between exhaust and inlet air.

Desiccant (silica gel) captures moisture from the air (Fig. 1a). Then, the moisture in the desiccant is desorbed by microwaves (Fig. 1b). The key feature of microwaves is that they can oscillate water molecules and desorb from the adsorbent's surface (silica gel). The lab-scale pilot microwave dehumidification system is illustrated in Fig. 7a, and its schematic diagram is shown in Fig. 7b**.** A microwave generator (Fricke und Mallah, Germany) was used to generate the microwaves. Frame and equipment were grounded with protective grounding to prevent users from high voltage electrical hazards. Two modes were considered: the mode without heat recovering and the mode with heat recovering from outlet air. Temperatures and differential pressure readings were logged continuously by software Labview and Agilent 34970A for both modes. The desiccant wheel rotating motor speed was set to the desired value, running only during desorption. Figure 7b demonstrates a setup diagram. The study performed the following procedure without heat recovery: Air damper-1 and air damper-3 were opened, and air damper-2 and air damper-4 were closed, letting the air bypass the heat-recovering device. Then, the honeycomb-structured desiccant wheel was saturated with moisture at constant relative humidity and temperature at a regular airflow rate until the inlet and outlet temperatures were the same: the same temperature and humidity indicated equilibrium conditions. Consequently, microwaves were switched on for the preset time and preset power from the control panel; the desorption process finishes when the outlet humidity ratio becomes lower than the inlet humidity ratio. Mode with heat recovery is similar to mode without heat recovery; When the inlet and outlet temperatures became the same, air damper-1 and air damper-3 were closed, and air damper-2 and air damper-4 were opened to recover heat from outlet air.

## Supplementary Information

Supplementary Information 1.

Supplementary Information 2.

## Data Availability

The presented data is available from the corresponding author on reasonable request.
