# Supplementary material for: Innovative solid desiccant dehumidification using distributed microwaves
Source: Sci Rep. 2023 May 6;13:7386. doi: 10.1038/s41598-023-34542-9 (PMC10164158; doi:10.1038/s41598-023-34542-9)
Supplement: Supplementary file 2 — Supplementary Information 2. [file 41598_2023_34542_MOESM2_ESM.docx]

Supplementary material for paper “Innovative solid desiccant dehumidification using distributed microwaves”

**Doskhan Ybyraiymkul**^1,*^**, Qian Chen**^2^**, Muhammad Burhan**^1^**, Faheem Hassan Akhtar**^3^**, Raid AlRowais**^4^**, Muhammad Wakil Shahzad**^5^**, M Kum Ja**^1^**, and Kim Choon Ng**^1,*^

^1^ Water Desalination and Reuse Center, BESE Division, King Abdullah University of Science and Technology, Thuwal, 23955, Saudi Arabia

^2^Institute of Ocean Engineering, Tsinghua University, Beijing, 100190, China

^3^Department of Chemistry & Chemical Engineering, Lahore University of Management Sciences, Lahore, 54792, Pakistan

^4^Civil Engineering Department, Jouf University, Jouf, 72388, Saudi Arabia

^5^Mechanical & Construction Engineering Department, Northumbria University, Newcastle Upon Tyne, NE7-7XA, UK

[^*^doskhan.ybyraiymkul@kaust.edu.sa](mailto:*doskhan.ybyraiymkul@kaust.edu.sa)

*Experimental setup*

Measuring devices and their accuracy are listed in Table 1. Uncertainties of the logged data were analyzed according to guidance [1].

Table 1. Sensors and their accuracy

| Parameter | Measuring device | Manufacturer | Range | Error |
| --- | --- | --- | --- | --- |
| Differential pressure | Differential pressure transducer Model 264 | Setra | 0-250 Pa | ±0.25% FS |
| Air temperatures | Pt100 RTD | Omega | -20~350 °C | ±0.1 °C |
| Desiccant temperature | Infrared temperature sensor OS-MINIUSB | Omega | 0~250 °C | ±1 °C |
| Electrical power | Power meter PowerLogic PM5110 | Schneider Electric | 3-30 kW | ± 0.5% |

*Experimental procedure*

Two cases were considered: the case without heat recovering and the case with heat recovering from outlet air. Temperatures and differential pressure readings were logged continuously by software Labview and Agilent 34970A for both cases. Desiccant wheel rotating motor speed was set to the desired value, and it was running only during desorption. Figure 1 shows procedure that was performed in the study without heat recovery:

1. Air damper-1, air damper-3 were opened, and air damper-2, air damper-4 were closed, letting the air bypass the heat recovering device.
2. Honeycomb structured desiccant wheel was saturated with moisture at constant relative humidity and temperature at a regular airflow rate until the inlet and outlet temperatures were the same. The same temperature and humidity show equilibrium conditions.
3. Microwaves were switched on for the preset time and preset power from the control panel;
4. The desorption process finishes when the outlet humidity ratio becomes lower than the inlet humidity ratio.
5. The Absorption process goes for a preset time.

Case with heat recovery is similar to case without heat recovery; only the second point is different. When the inlet and outlet temperatures became the same, air damper-1, air damper-3 were closed, and air damper-2, air damper-4 were opened to recover heat from outlet air.

**Figure 1.** Operation procedure of the experiments.

*Complex permittivity calculation for wheel*

Complex permittivity is compounded of real and imaginary parts:

${\varepsilon'}_{r,eff}=f_{op}{\varepsilon^{'}}_{r,air}+f_{cd}{\varepsilon^{'}}_{r,cd}$ (1)

${\varepsilon"}_{r,eff}=f_{cd}{\varepsilon"}_{r,cd}$ (2)

Microwave energy penetration and its consumption strongly depend on the effective complex permittivity of the desiccant wheel. Effective complex permittivity depends on adsorption uptake (water vapor loading), so it must be obtained for different uptake values.

*References*

[1] “Evaluation of measurement data — Guide to the expression of uncertainty in measurement.” JCGM, 2008. [Online]. Available: https://www.bipm.org/documents/20126/2071204/JCGM_100_2008_E.pdf/cb0ef43f-baa5-11cf-3f85-4dcd86f77bd6
